# Improving 3D ultrasound prostate localisation in radiotherapy through increased automation of interfraction matching

**DOI:** 10.1016/j.radonc.2020.04.044

**Published:** 2020-08

**Authors:** Alexander Grimwood, Hassan Rivaz, Hang Zhou, Helen A. McNair, Klaudiusz Jakubowski, Jeffrey C. Bamber, Alison C. Tree, Emma J. Harris

**Affiliations:** a Division of Radiotherapy and Imaging, The Institute of Cancer Research and Royal Marsden Hospital Trust, Sutton, UK; b Department of Electrical and Computer Engineering, Concordia University, Montreal, Canada; c Department of Medical Physics, University of Silesia, Chorzów, Poland

**Keywords:** Image-guided radiotherapy, Ultrasonography, Radiotherapy setup error, 3D imaging, Prostate cancer, Automation

## Abstract

**Background and purpose:**

Daily image guidance is standard care for prostate radiotherapy. Innovations which improve the accuracy and efficiency of ultrasound guidance are needed, particularly with respect to reducing interobserver variation. This study explores automation tools for this purpose, demonstrated on the Elekta Clarity Autoscan®. The study was conducted as part of the Clarity-Pro trial (NCT02388308).

**Materials and methods:**

Ultrasound scan volumes were collected from 32 patients. Prostate matches were performed using two proposed workflows and the results compared with Clarity’s proprietary software. Gold standard matches derived from manually localised landmarks provided a reference. The two workflows incorporated a custom 3D image registration algorithm, which was benchmarked against a third-party application (Elastix).

**Results:**

Significant reductions in match errors were reported from both workflows compared to standard protocol. Median (IQR) absolute errors in the left–right, anteroposterior and craniocaudal axes were lowest for the Manually Initiated workflow: 0.7(1.0) mm, 0.7(0.9) mm, 0.6(0.9) mm compared to 1.0(1.7) mm, 0.9(1.4) mm, 0.9(1.2) mm for Clarity. Median interobserver variation was ≪0.01 mm in all axes for both workflows compared to 2.2 mm, 1.7 mm, 1.5 mm for Clarity in left–right, anteroposterior and craniocaudal axes. Mean matching times was also reduced to 43 s from 152 s for Clarity. Inexperienced users of the proposed workflows attained better match precision than experienced users on Clarity.

**Conclusion:**

Automated image registration with effective input and verification steps should increase the efficacy of interfraction ultrasound guidance compared to the current commercially available tools.

## Highlights

• Automated matching improves accuracy and precision of ultrasound guided radiotherapy.
• Image registration software lowers setup errors and sonography training requirements.
• Spatial regularisation improves registration algorithm performance.

Accurate image guidance is essential to minimise setup errors and facilitate reduced margins in prostate radiotherapy. This is especially the case for ultrahypofractionation which may become standard within a few years. However, streamlined workflows are required to reduce interobserver variability in matching and to improve departmental efficiency.

Ultrasound imaging (US) is non-invasive, non-ionising, cost-effective and allows for direct visualization of the prostate and surrounding tissues in 4D (serial 3D imaging). Systems can be used for interfraction and intrafraction motion management [1], [2] and some radiotherapy departments are using ultrasound as their standard image guidance method for prostate cancer. The Clarity Autoscan system uses a 3D transperineal ultrasound (TPUS) probe and provides continuous imaging of the prostate for intrafraction motion estimation [3], [4]. The Clarity system uses manual comparison of an image acquired at simulation with one acquired prior to treatment to calculate the couch shift necessary to correct for interfraction motion. This requires the radiation therapist to scroll back and forth through the 3D volume in two or three of the axial, sagittal and coronal planes, iteratively adjusting a matching contour (the reference position volume (RPV) contour), which can be time-consuming and requires significant familiarity with ultrasound image interpretation. Ultrasound is a user-dependent and observer-dependent modality, leading to variations in image quality and image interpretation, which further contributes to uncertainty in the estimated interfraction motion [5].

Automating the matching of simulation and treatment TPUS images would reduce the complexity of US-guided interfraction motion correction and could improve precision. Similar to commercial image-guided radiotherapy software, automated match results should be displayed to the operator for visual inspection and approval prior to couch correction [6]. A quantitative measure of match quality could also be developed to assist the user in deciding whether the match is acceptable, a step commonly used after automated registration of CBCT images [7]. This study examines two possible clinical workflows with differing levels of automation, comparing their overall speed and precision to provide an insight into how the integration of such tools can improve the standard of care in US image-guided radiotherapy.

## Materials and methods

An application was developed to automate interfraction matching of a manually defined prostate reference positioning volume (RPV) in TPUS scans acquired using Elekta Clarity Autoscan (Elekta AB., Sweden) [4], [8]. The application was used to simulate two potential workflows; both of which were tested against the current clinical protocol using retrospective data. A custom 3D template matching algorithm was developed for the application and benchmarked against Elastix, an established third party image registration software [9], [10]. A training dataset was used for development, optimisation and benchmarking. The workflows were then validated on a separate test dataset to demonstrate interpatient generalisability, where larger variations in anatomical appearance and image quality are expected.

### Patients

Patients referred for radical radiotherapy to the prostate were recruited to the Clarity-Pro trial (NCT02388308), approved by the Surrey and SE Coast Regional Ethics Committee, UK [15]. From 42 patients a random selection of 32 were analysed for this study. All trial patients received CBCT image guidance in line with the clinical standard of care. Ultrasound scans were also acquired at simulation and during CBCT acquisition as described below. Assuming all match errors are normally distributed with a 1.0 mm standard deviation and no systematic bias, the population size results in an error measurement precision with a ±0.5 mm 95% confidence interval and a standard error of ±0.3 mm [11].

### Clarity image acquisition

3D ultrasound scans were obtained from the 32 selected patients. Volumetric data was recorded using the Clarity Autoscan probe, which is optically tracked to enable 3D image reconstruction in DICOM room coordinates. At simulation, a CT scan was acquired before realigning the patient to the room lasers and acquiring a reference US scan.

Treatment planning was conducted using Pinnacle (Philips Healthcare, Amsterdam, Netherlands), after which simulation CT and planning contours were imported into the Clarity workstation. A trained operator ensured the reference US and CT scans were co-registered before manually contouring a prostate US RPV, aided by the CT and clinical treatment volume (CTV) contour. The CTV could not directly be used as the US RPV, because it often incorporated the seminal vesicles and because the CT voxel size was significantly larger compared to US.

For each fraction, the patient set up from simulation was reproduced. A guide ultrasound scan was acquired by a trained radiation therapist prior to the patient being treated on a conventional Elekta Synergy linac using CBCT image guidance. The ultrasound probe remained fixed in place throughout radiation delivery.

### Clarity matching

Prostate matching was performed offline by three experienced observers (two physicists and one radiation therapist) following the standard clinical workflow in the Clarity Guide Review software. Observers viewed reference and guide scans side by side in sagittal, coronal and transverse planes. The RPV contour was superimposed over the reference ultrasound scan and the user placed an identical guidance positioning volume (GPV) contour in the same position on the guide scan.

### Gold standard matches

For each fraction, up to five landmarks visible both within the RPV and guide image were manually localized. These landmarks included calcifications that were clearly visualized in some ultrasound scans, acting as endogenous fiducial markers [12]. Matches were defined as the mean landmark shift and a gold standard result calculated to be the mean landmark match from three experienced observers. Any fraction with an interobserver difference greater than 5 mm was repeated independently by all three observers up to two times to reduce uncertainties. Gold standard results were used to evaluate the accuracy of all other match methods described in this study.

### Template matching algorithm

A dedicated registration algorithm was developed, because third party registration tools were either computationally slow, or were too sensitive to variations in image quality between scans caused by changes in probe and patient position. A correlation based algorithm was chosen due the technique's ability to accurately estimate motion in clinical ultrasound images, as reported previously by O'Shea et al. and Shams et al. [7], [13]. Algorithm results were validated against matches derived from manually identified endogenous fiducial prostate landmarks and also against the current Clarity matching software. The Clarity software did not quantify prostate rotations and the algorithm likewise only estimated translations.

Spatial regularization methods were used to ensure the algorithm was robust to images containing few discernible features or large variations in image quality caused by patient and probe motion. A detailed description of the algorithm is given in Supplementary Materials 1 and the code is available upon request.

### Prostate matching workflows

**Fig. 1:**
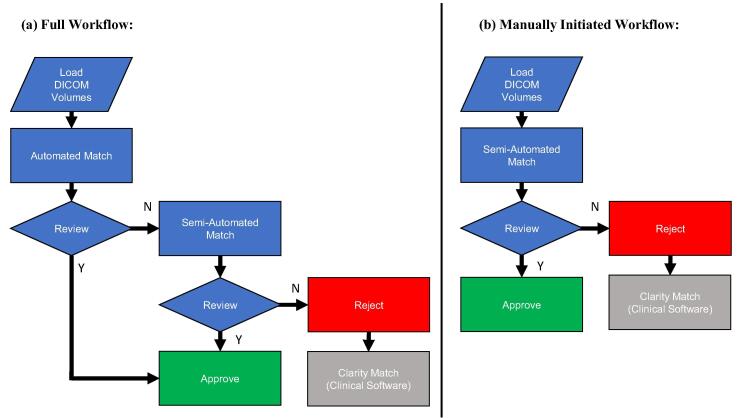
Flowcharts for the two semi-automated matching workflows: (a) Full workflow and (b) Manually Initiated workflow.

Two workflows – Full and Manually Initiated (Fig. 1) – were devised to examine the best way of clinically implementing the algorithm with manual inspection steps of both scans and match results. The Full workflow comprised three matching methods: (1) automated matching – where the previously described algorithm was used; (2) semi-automated matching – for which the user manually located the approximate prostate location by placing a small rectangular search window (15 pixels larger than the RPV) around it; (3) Clarity matching – manual matches performed on the Clarity Guide Review software. An automated match was performed and reviewed. If unsatisfactory, a semi-automated match was performed. If this match was also rejected, the user resorted to a manual Clarity match.

For the Manually Initiated workflow, only the semi-automated matching and Clarity matching steps were performed. For both workflows, the output was recorded as the first approved match, or the Clarity match in case of rejection.

At review, matches were displayed to the user by overlaying RPV and GPV contours on their respective reference and guide image volumes. A match summary was also generated to aid visual assessment of match quality. It comprised central sagittal and coronal planes through the positioning volumes with contour overlays and an accompanying correlation map (Supplementary Materials 2).

Match results and observer decisions were collected for every image pair using all three matching methods. The two workflows were retrospectively simulated for one experienced and two inexperienced observers based upon their review decisions. Due to patient confidentiality considerations, all Clarity match results were performed by three experienced clinical staff and the mean match results used for all observers. The inexperienced observers underwent training, comprising provision of an instruction manual and a practice session with experienced staff using the training dataset. A gap of at least two weeks was imposed between observers performing matches using the automated and semi-automated methods to restrict their familiarisation with the data. Review decisions from each observer were automatically recorded and used to recreate the workflows. Match and review timings were also recorded for all three methods to produce estimates of total workflow times.

### Analysis and application development

Match errors, E, were calculated as the relative difference between match result and the gold standard. Translational registrations using Elastix software provided a third-party comparator to evaluate algorithm accuracy on the training dataset by comparing error distributions, absolute error, $\left|E\right|$, medians and interquartile ranges. Correlation coefficients, C, between each method and the gold standard were calculated to measure how comparable the results were. Both methods were optimised prior to evaluation as described in Supplementary Materials 3.

Workflow evaluation was conducted on the test dataset. Error means, standard deviations, and ranges were compared. Timings, t, for individual matches and the entire workflows were recorded, as were rejection rates for each match method. Finally, for Clarity and both workflows, interobserver variation (IOV) was quantified as the maximum difference between observers for each fraction. Matlab (MathWorks Inc., USA) running on an Intel 2.8 GHz Xeon CPU with 16 GB RAM was used to write the algorithm, develop the application and perform all subsequent analyses.

## Results

For each selected patient, a reference scan was acquired during simulation, and guide scans from five fractions were collected producing 160 reference-guide scan pairs. The training dataset comprised 100 scan pairs from 20 patients. The remaining 60 scan pairs from 12 patients formed the test dataset.

Using the training dataset, accuracy was assessed against the gold standard. The algorithm and Elastix both produced significant accuracy and precision improvements over Clarity according to statistical testing of median absolute errors, $\left|E\right|$, (Mann–Whitney *U*: p<0.05) and dispersion in E (Ansari–Bradley: p<0.05). Comparable errors were observed between the algorithm and Elastix, $\left|E\right|$, (Mann–Whitney *U*: p>0.05) and E (Ansari–Bradley: p>0.05). Error distributions were confirmed non-normal using *t*-tests, although there was no indication of bias beyond outliers in the error distributions with all mean errors, $\overset{¯}{E}\leq 1.1$ mm (2 pixels).

Clarity absolute error median and interquartile ranges for the Left-Right (LR), Anterior-Posterior (AP) and Superior-Inferior (SI) axes were ${\left|E\right|}_{\mathrm{median}}\left(IQR\right)=1.5\left(1.8\right)\;\text{mm,}\;1.0\left(1.4\right)\;\text{mm,}\;1.1\left(1.8\right)\;\text{mm}$. For Elastix, ${\left|E\right|}_{\mathrm{median}}\left(IQR\right)=0.6\left(0.9\right)\;\text{mm, 0}.7\left(1.4\right)\;\text{mm,}\;0.7\left(1.2\right)\;\text{mm}$. And for the algorithm, ${\left|E\right|}_{\mathrm{median}}\left(IQR\right)=0.7\left(0.8\right)\;\text{mm,}\;0.6\left(1.0\right)\;\text{mm, 0.6}\left(1.1\right)\;\text{mm}$.

All methods were significantly correlated to the gold standard with p<0.05 in every axis for all matching methods. Algorithm correlation was strongest, with coefficients C= 0.87, 0.93 and 0.92 (LR, AP, SI respectively). For Clarity C= 0.78, 0.91 and 0.85. For Elastix, C= 0.87, 0.68 and 0.22. Poor Elastix matches in four fractions from a single patient where $\left|E\right|<19$ mm (AP) and $\left|E\right|<30$ mm (SI) resulted in weaker correlations (Supplementary Materials 3). An inspection of the patient images found anatomical changes caused by rectal filling at simulation not observed in subsequent treatment images.

Elastix produced the largest error range: -29.4mm≤E≤6.8mm across all axes, while the algorithm exhibited the smallest error range: -7.4mm≤E≤6.1mm. However mean calculation times were longer for the algorithm: ${\overset{-}{t}}_{\mathrm{algo}}=113\;\text{s}$, ${\overset{-}{t}}_{\mathrm{elastix}}=58\;\text{s}$.

**Fig. 2:**
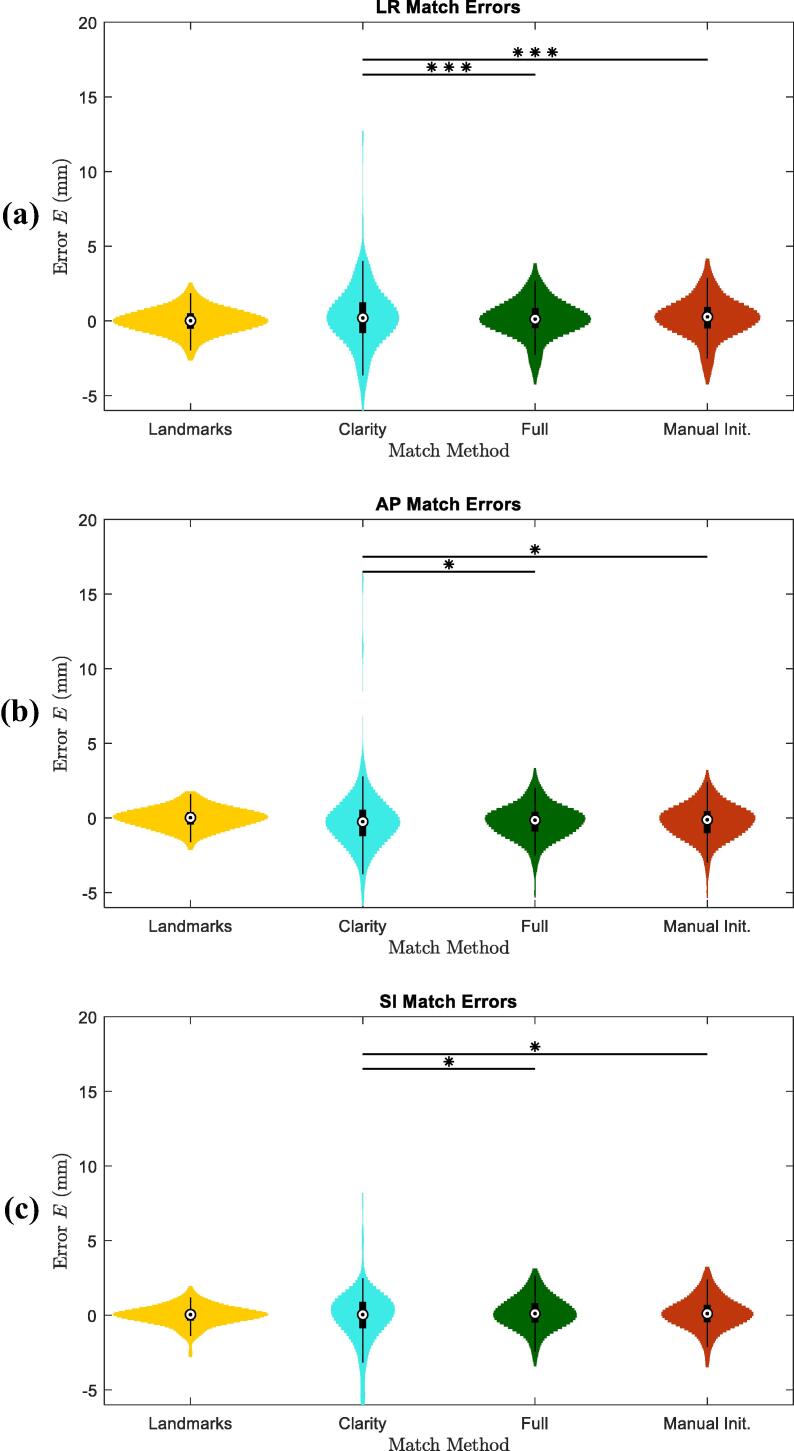
Match error (E) violin distributions from all three observers across Clarity, Full and Manually Initiated workflows with Manual Landmark match errors for reference. Significance symbols are shown for paired *F*-tests between Clarity and algorithm workflows.

The two matching workflows were assessed on the test dataset (Table 1). Both workflows produced significant error improvements compared to Clarity according to paired *t*-tests (p<0.05) and paired F-tests (p<0.05). The same tests showed the results arrived at by both workflows exhibited statistically equivalent errors (p>0.05 in all cases). All error distributions were confirmed normal using *t*-tests and are displayed in Fig. 2. As shown in Table 1, Clarity match accuracy was hampered by outlying errors as large as ±16.0 mm that were not evident in either Full or Manually Initiated workflows, where absolute axial errors were reduced to within ±5 mm. No indication of bias was found, demonstrated by mean errors $E_{\mathrm{mean}}$ within ±0.5 mm for Clarity and for both workflows. The workflows improved precision, reducing axial error standard deviations $E_{\mathrm{std}}$ from ≤2.1 mm using Clarity, to ≤1.4 mm using the Full workflow and ≤1.3 mm using the Manually Initiated workflow.

**Table 1:** Test dataset match errors (E) in each patient axis, absolute error medians (${\left|E\right|}_{med}$), interquartile ranges (${\left|E\right|}_{IQR}$) and match times (t) for: manually selected landmarks, the Clarity workflow, Full workflow and Manually Initiated workflow. Landmark match times were not recorded.

	Landmarks			Clarity			Full			Manually Initiated		
	LR	AP	SI	LR	AP	SI	LR	AP	SI	LR	AP	SI
$E_{\mathrm{mean}}$(mm)	–	–	–	0.3	−0.2	−0.1	0.1	−0.3	0.1	0.2	−0.2	0.1
$E_{\mathrm{std}}$(mm)	0.8	0.7	0.7	2.1	2.1	1.8	1.3	1.1	1.1	1.4	1.1	1.1
$E_{\min}$(mm)	−2.4	−1.9	−2.6	−5.6	−5.5	−6.0	−3.9	−5.0	−3.1	−3.9	−5.0	−3.2
$E_{\max}$(mm)	2.5	1.6	1.9	12.5	16.0	7.8	3.7	3.0	3.0	4.0	3.0	3.0
${\left|E\right|}_{\mathrm{med}}$(mm)	0.5	0.4	0.3	1.0	0.9	0.9	0.7	0.6	0.7	0.7	0.7	0.6
${\left|E\right|}_{\mathrm{IQR}}$(mm)	0.6	0.6	0.5	1.7	1.4	1.2	1.0	0.9	0.9	1.0	0.9	0.9
$t_{\mathrm{mean}}$(s)	–			152			131			43		
$t_{\min}$(s)	–			16			24			15		
$t_{\max}$(s)	–			308			308			136

The three observers’ rejection rates for automated matches were: 24%, 21% and 11%, with the two inexperienced observers recording significantly higher rejection rates. Semi-automated matches produced lower, more equitable rejection rates of: 2%, 7% and 5% respectively. The Manually Initiated workflow was also significantly quicker than both the Full workflow and Clarity with mean match times, $\overset{-}{t}=$ 43 s, 131 s and 152 s respectively (Table 1).

**Fig. 3:**
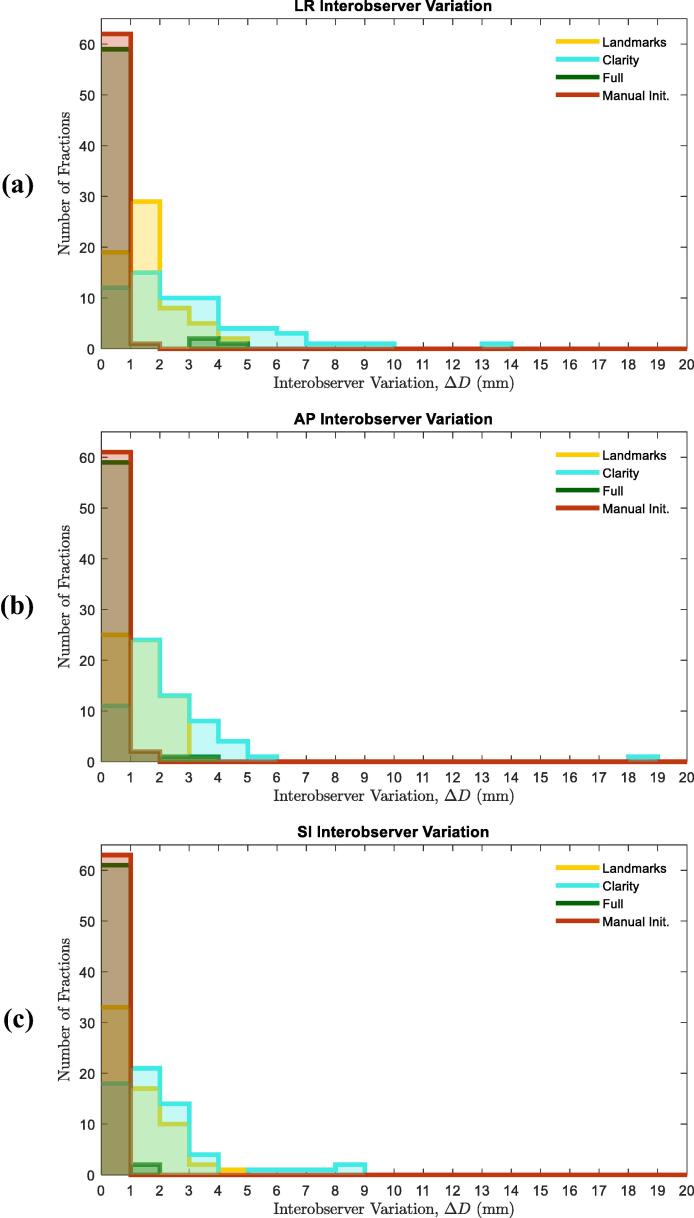
Histograms of interobserver variation (ΔD) in match results for Clarity, Full and Manually Initiated workflows, with Manual Landmark matches for reference.

Match uncertainties arising from interobserver variation were almost completely suppressed by the workflows as shown in Table 2 and Fig. 3. The median IOV was ≪0.01 mm in all axes for both workflows, conversely the largest Clarity median IOV was 2.2 mm in the LR axis.

**Table 2:** Interobserver match variation (IOV) for the Clarity, Full and Manually Initiated Workflows with manual landmark matching for reference.

*IOV* (mm)	Landmarks			Clarity			Full			Manually Initiated		
	LR	AP	SI	LR	AP	SI	LR	AP	SI	LR	AP	SI
25%	0.9	0.7	0.6	1.1	1.2	0.9	0.0	0.0	0.0	0.0	0.0	0.0
50%	1.5	1.3	1.0	2.2	1.7	1.5	0.0	0.0	0.0	0.0	0.0	0.0
75%	2.0	1.9	1.8	3.8	2.6	2.4	0.0	0.0	0.0	0.5	0.5	0.5
IQR	1.1	1.3	1.2	2.7	1.4	1.5	0.0	0.0	0.0	0.5	0.5	0.5

## Discussion and conclusions

This study suggests automated matching algorithms can improve the accuracy of ultrasound-guided prostate radiotherapy, especially when incorporated into a broader workflow with simple manual input and verification steps. Such a workflow may allow the technique to become more widely used clinically. Our results thus demonstrate how to raise the current standard of care for ultrasound guided radiotherapy. Furthermore, the introduction of TPUS imaging was primarily to enable intrafraction prostate motion monitoring, which has been shown to have high accuracy and precision, implementing an interfraction guidance protocol would provide a complementary application [3], [14]. Some guidance technologies, such as imaging implanted fiducial markers with planar kV, or CBCT may confer an accuracy advantage, however ultrasound provides superior soft tissue contrast and the ability to image continuously without increased radiation dose [15]. The accuracy of markerless CBCT is reported to be comparable to current ultrasound guidance techniques, with many centres avoiding marker implantation due to the increased need for resources and associated risk of infection [16]. Furthermore, with further development, ultrasound may be sufficiently accurate to enable daily adaptive replanning on a range of radiotherapy systems [17].

Workflows incorporating an image registration algorithm significantly improved match accuracy compared to Clarity. Match error ranges and standard deviations were significantly reduced, as was interobserver variation. Training staff to interpret ultrasound images is a recognised challenge, especially for departments where resources are often stretched [5], [18]. Our proposed workflows could reduce time-pressure and training burdens for radiotherapy practitioners, as demonstrated by the effectiveness of inexperienced users operating our software. The proposed workflows also profoundly reduced interobserver variation, which has been implicated in poor agreement between US and CBCT by Fargier-Voiron et al. [8], [19]. The same group reported variations in TPUS probe pressure significantly impacted prostate motion, with repercussions for treatment quality [8].

Match times were comparable to Clarity when using the Full workflow ($t_{\mathrm{mean}}=151\;\text{s}$ and 132s respectively) and significantly faster using the Manually Initiated workflow (43s). Users had to assess prostate location and any indications of significant morphological changes more closely when performing a Manually Initiated match compared to the automated method, because they were tasked with manually positioning a search window. Review times subsequently improved and rejection rates decreased. Automated match rejections were 11% for an experienced observer and <20% for inexperienced observers, but decreased to ≤7% for all observers using Manually Initiated matching, possibly due to greater confidence in the semi-automated match result arising from a more thorough examination of the images.

A subset of images from two patients were consistently rejected by all observers. Variations in prostate appearance and geometry were identified between these scans despite the requirement in our scan protocol for maintaining good image quality, minimising probe-patient contact and assessing the penile bulb for consistency. These variations likely resulted in dissatisfaction with the rigid registration results. A robust deformable registration algorithm could elicit greater match confidence, but requires sophisticated treatment plan adaptation to the deformed target volume. Even though all Clarity scans are recorded with the probe fixed in place, locating the optimal scan position and acquisition parameters requires significant user involvement. Other studies have sought to automate probe set up, which would further reduce registration errors [17]. In its current form, Clarity lacks the ability to assess prostate rotation and this study suggests the system is not yet suitable for patients where such motion is clearly observed. For this reason, we suggest the proposed workflow should be used in conjunction with other image guidance techniques, such as CBCT, for cases where large rotations or deformations are observed in the review step of the workflow.

The gold standard was derived from a consensus match of up to five common prostate landmarks, with ≤3 landmarks recorded for 17% of matches and observer variations up to ±2.6 mm from the mean. This carried an inherent uncertainty and was a compromise in the absence of a reliable ground truth. Other studies have used a reference imaging modality, such as CBCT with markers, to assess ultrasound prostate localisation accuracy. While the correlation between the different modalities can be used to assess relative performance, the inherent uncertainties of the reference method often remain unquantified and may implicitly degrade the perceived accuracy of ultrasound guidance. Scale Invariant Feature based registration was also investigated for this study, however the sparsity of common features or landmarks produced poor results in the presence of even relatively minor motion, often resulting in divergence during optimisation. The lack of common features was attributed to significant changes in image quality and poor spatial resampling of US image volumes from a stack of B-scans in polar coordinates onto a cartesian grid in room space. Although the presence of echogenic features should have aided the registration algorithms, an assessment of registration errors relative to the mean number of features identified found no clear relationship. This was likely due to the over-riding influence of other factors such as variations in the appearance of features between scans. The gold standard also could not adequately describe rotational, affine or deformable prostate motion. Furthermore, the increased computational cost would have slowed match times and limited the workflow’s usability.

Previous intramodality registration algorithms have been reported for patient positioning in prostate RT using transabdominal ultrasound [20], [21]. Kaar et al. reported a mean Euclidean error and standard deviation ${\overset{-}{E}}_{\mathrm{euc}}=$ 3.0(1.5) mm [20]. Similarly, Presles reported ${\overset{-}{E}}_{\mathrm{euc}}=$ 3.5 mm with $E_{\mathrm{std}}=$ 1.7 mm, 2.6 mm and 2.4 mm in LR, AP and SI axes [21]. By comparison, our Semi-Automated workflow exhibited smaller errors: ${\overset{-}{E}}_{\mathrm{euc}}=$ 1.8(1.0) mm and $E_{\mathrm{std}}=$ 1.3 mm, 1.1 mm, 1.1 mm.

Future studies will investigate the use of deformable registration methods in conjunction with the polar US scan volumes to improve match accuracy and intermodality registration. Technical support from Elekta is needed to integrate automated matching software with a TPUS system for online testing and validation. Routine clinical implementation will also require industrial support and regulatory approval.

Two workflows incorporating automated image registration with varying levels of manual input were devised, tested and compared to the current standard practice of manually matching volumetric ultrasound scans. A registration workflow incorporating manual initialisation and verification was found to be superior to automated registration alone. Such a workflow would improve efficacy of interfraction prostate localisation in ultrasound guided radiotherapy compared to standard practice.

## Sources of support

NHS funding was provided to the National Institute for Health Research Biomedical Research Centre at the Royal Marsden Hospital and The Institute of Cancer Research. This research is also supported by 10.13039/501100000289Cancer Research UK under programs C33589/A19727 and C20892/QA23557, and by the 10.13039/501100000270NERC Discovery Grant RGPIN04136.

## Declaration of Competing Interest

The authors declare the following financial interests/personal relationships which may be considered as potential competing interests: A.T. reports support from Elekta as a clinical research fellow working on other projects (not related to this project) and personally has received honoraria and travel grants from Elekta to cover meeting attendance. J.B. reports grants from Cancer Research UK, from the Engineering and Physical Sciences Research Council, from the Biotechnology and Biological Sciences Research Council, and from the National Institute for Health Research, outside the submitted work, and previously acted as a consultant for Elekta, although not during the period of work for this study.
