# Supplementary material for: Improving 3D ultrasound prostate localisation in radiotherapy through increased automation of interfraction matching
Source: Radiother Oncol. 2020 Aug;149:134–41. doi: 10.1016/j.radonc.2020.04.044 (PMC7456791; doi:10.1016/j.radonc.2020.04.044)
Supplement: Supplementary data 1 [file mmc1.docx]

**Supplementary Materials 1**

A patch-based algorithm incorporating normalised cross correlation (NCC) and spatial regularization was developed to be robust to images containing few discernible features or large variations in image quality caused by patient and probe motion. Figure 1 is an illustration of the processing steps. During standard clinical workflow, a reference positioning volume (RPV) was manually contoured onto the reference ultrasound scan, denoting the prostate. For the algorithm, the RPV was used to define a template, which was compared to subvolumes in the guide image in order to find the best match. The RPV template mask was grown by 4 pixels to ensure incorporation of the prostate boundary. The template was then divided into subvolumes, referred to as patches. For each patch, $r$, the room coordinates were derived from optical tracking data and a search volume from the guide image, $g$, at the same location was assigned. A search size margin of 34 pixels (19.6 mm) around the patch was used, which accommodated the largest prostate shift observed in the training dataset.

The NCC for a single patch can be expressed as:

| $C\left( i \right)=\frac{\sum_{j=1}^{n} \left( r_{j}-\bar{r} \right)\left( g_{j}(i)-\bar{g(i)} \right)}{\left( \sum_{j=1}^{n} \left( r_{j}-\bar{r} \right)^{2}\sum_{j=1}^{n} \left( g_{j}(i)-\bar{g(i)} \right)^{2} \right)}$ | (1) |
| --- | --- |

where *r* and *g* comprise $n$ pixels and have respective means $\bar{r}$ and $\bar{g}$. A patch at position $i$ within the search volume produces a normalised correlation coefficient at that point, $C(i)$. Reference and guide volumes were resampled to form a Gaussian image pyramid with $L$ levels, where each level is a Gaussian filtered image volume downsampled by a factor, *L*. Intensity gradient images, $G$, for all three axes were also produced so each level constituted one intensity and three gradient reference-guide pairs: *I*, $Gx=\frac{\partial I}{\partial x}$, $Gy=\frac{\partial I}{\partial y}$, $Gz=\frac{\partial I}{\partial z}$. NCC of all image pairs from each pyramid level were resampled and combined to form a single correlation map:

| $\boldsymbol{C}=\frac{1}{LN}\sum_{j=1}^{LN} \left( C_{L0}+{Cx}_{L0}+{Cy}_{L0}+{Cz}_{L0}+\ldots C_{Ln}+{Cx}_{Ln}+{Cy}_{Ln}+{Cz}_{Ln} \right)$ | (2) |
| --- | --- |

where $\boldsymbol{C}$ is the mean correlation map from $N$ patches calculated at each level $L$. A Gaussian weighting function, $W$, with a standard deviation, $\sigma$, half the search window size was applied as a final regularization:

| $W\left( i \right)=\frac{1}{\sigma^{3}\left( 2\pi\right)^{3/2}}e^{\left( \frac{{-i}^{2}}{{2\sigma}^{2}} \right)}$ $Q(i)=W\left( i \right)\boldsymbol{C}(i)$ | (3) |
| --- | --- |
|  | (4) |

RPV matching was achieved by locating $\arg maxQ(i)$ in the guide search volume. A Fast 3D NCC algorithm using Fourier Domain convolution was adapted from Piotr Dollár’s MATLAB Toolbox to enable exhaustive interrogation of all search volumes within acceptable processing times [1].

**d)**

**e)**

**c)**

**b)**

**a)**

Figure 1.Registration process for a single pyramid level. The reference image template and guide search volume are defined (a). For each, intensity gradient images are produced (b). Template and search images are divided into patches, r and g respectively (c). Each patch pair is cross correlated, C, and the outputs combined to form a single spatially regularised correlation map, Q (d). RPV displacement is estimated from the correlation peak in Q (e).

References

1. Dollár, P. *Piotr's Computer Vision Matlab Toolbox (PMT)*. [cited 2019; Available from: <https://github.com/pdollar/toolbox>].
