# Supplementary material for: Improving 3D ultrasound prostate localisation in radiotherapy through increased automation of interfraction matching
Source: Radiother Oncol. 2020 Aug;149:134–41. doi: 10.1016/j.radonc.2020.04.044 (PMC7456791; doi:10.1016/j.radonc.2020.04.044)
Supplement: Supplementary data 2 [file mmc2.docx]

**Supplementary Materials 2**

**Application screenshots**

**
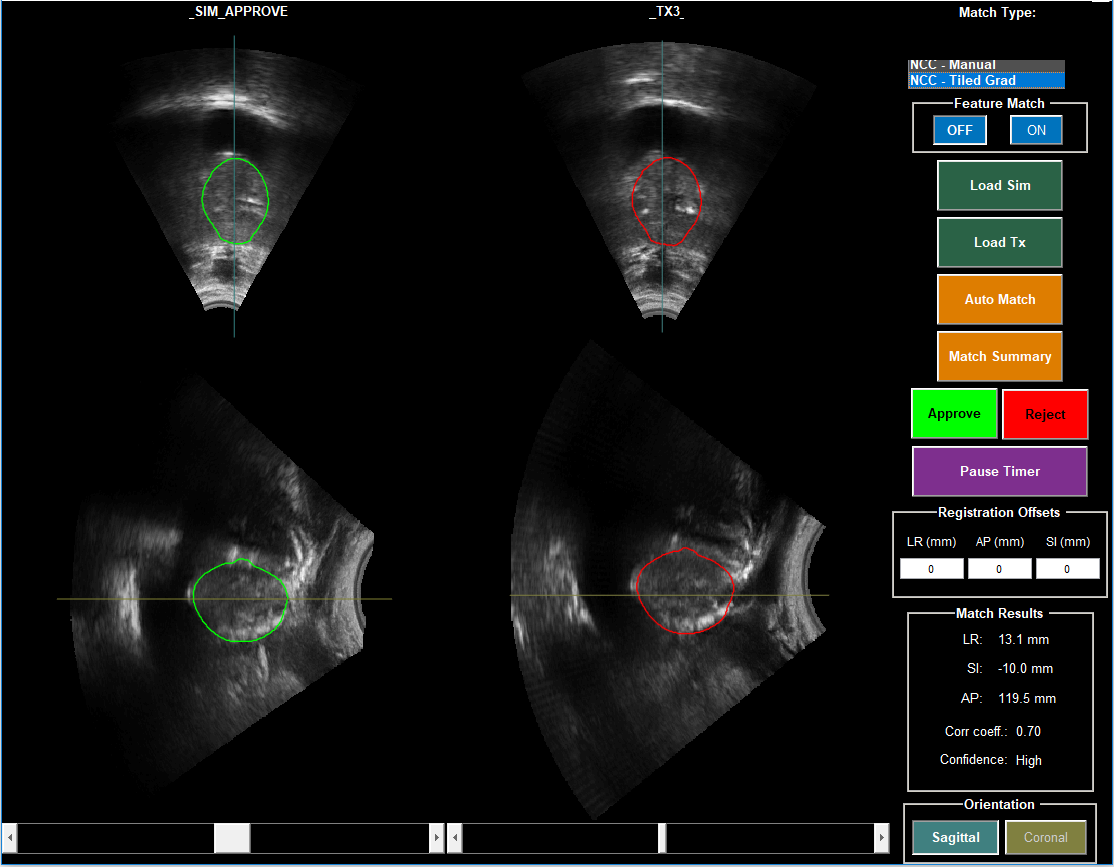
**

fig. 1 Application screenshot showing automated match result: GPV (red), RPV (green). The displayed AP shift includes a planning registration offset of 14.0 mm LR, -7.5 mm SI and 122.4 mm AP.


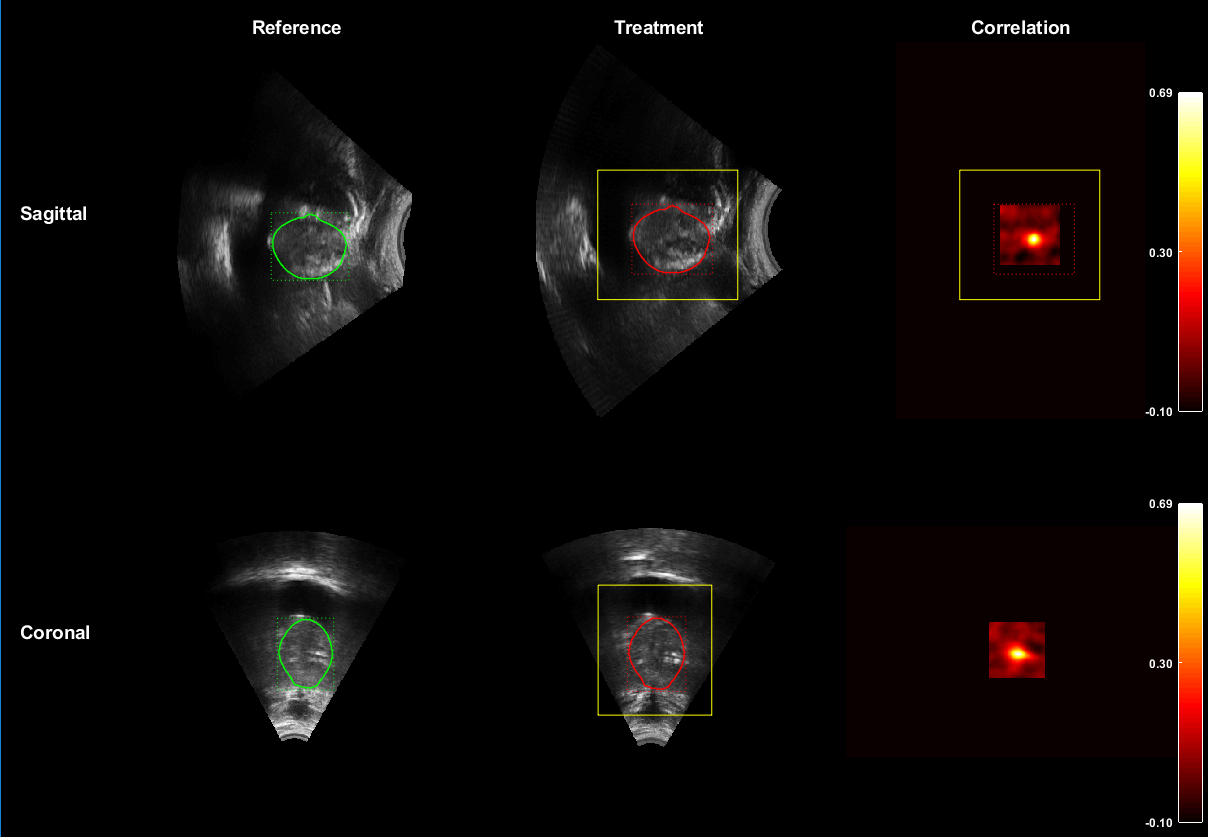


fig. 2. Match summary screen, depicting central planes through RPV and GPV and the associated correlation maps.

**
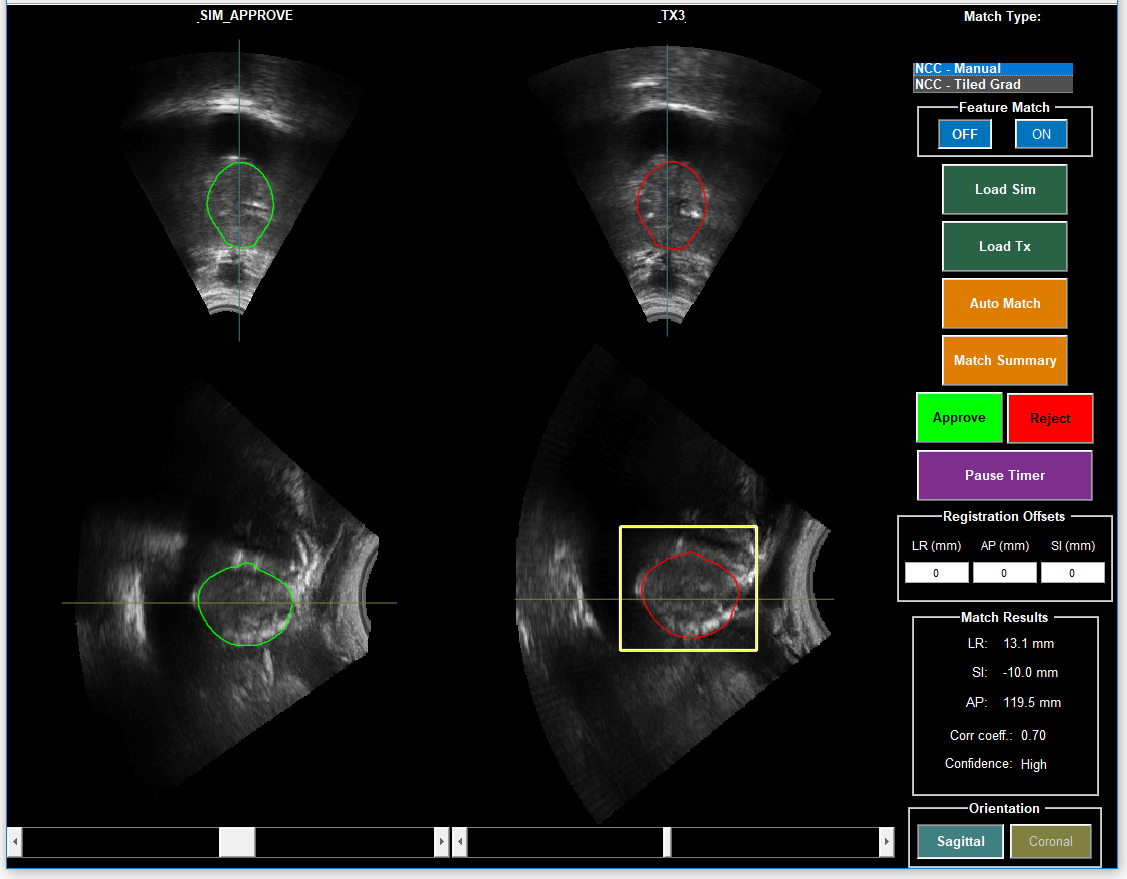
**

fig. 3. Semi-automated match, with manually positioned search region (yellow box). The displayed AP shift includes a planning offset of 14.0 mm LR, -7.5 mm SI and 122.4 mm AP.
