# Supplementary material for: Improving 3D ultrasound prostate localisation in radiotherapy through increased automation of interfraction matching
Source: Radiother Oncol. 2020 Aug;149:134–41. doi: 10.1016/j.radonc.2020.04.044 (PMC7456791; doi:10.1016/j.radonc.2020.04.044)
Supplement: Supplementary data 3 [file mmc3.docx]

**Supplementary Materials 3**

**Algorithm optimisation:**

A training dataset comprising 100 reference-guide pairs from 20 patients was used to assess the following parameters: choice of similarity metric, patch size and inclusion of gradient correlations. A simple optimisation was performed by evaluating which parameters minimised match error (*E*) mean, standard deviation and range in conjunction with mean processing time.

Normalised cross correlation (NCC) and normalized mutual information (NMI) with intensity bins ranging from 8 to 64 were tested against the gold standard (fig. 1). Patch sizes ranging from 25×25×25 pixels to 65×65×65 pixels were also tested for NCC (fig. 2).

The algorithm was validated against Elastix, which was configured to use an RPV template mask and calculate a linear transform. Elastix was optimised in the same fashion as the algorithm; parameters investigated were: similarity metric, pyramid levels, number of spatial samples and number of iterations.

Pyramids between 1 to 3 levels were used, while a spatial sample range of 50 to 50,000 and iteration range of 12 to 250 were explored. An advanced stochastic gradient descent optimiser was used in all cases. Configurations using Normalised cross correlation and Mattes Mutual information (with a range of bin sizes from 8 to 64) were both optimised and compared to the algorithm (fig. 3).

Finally, the optimised algorithm and Elastix were compared to the gold standard manual landmark matches and Clarity software (fig. 4).

**Results**

*Algorithm Optimisation:*

Normalised cross correlation exhibited higher accuracy for both the algorithm and Elastix. A patch size of 35×35×35 pixels suppressed match errors and a two level image pyramid maximised algorithm accuracy. For Elastix the following parameters were selected: 200 iterations, 40000 spatial samples, 3 pyramid levels and 32 histogram bins (for Mattes mutual information). The largest observed prostate shift in the training set was $<19$ mm and a search margin of 19.6 mm was subsequently chosen for all matches.

(b)

(a)

fig. 1 Boxplots depicting match errors (a) and processing times (b) for two different algorithm quality metrics: normalized cross correlation (NCC) and normalized mutual information (NMI).

fig. 2 Boxplots of match errors for NCC with differing patch (tile) sizes.


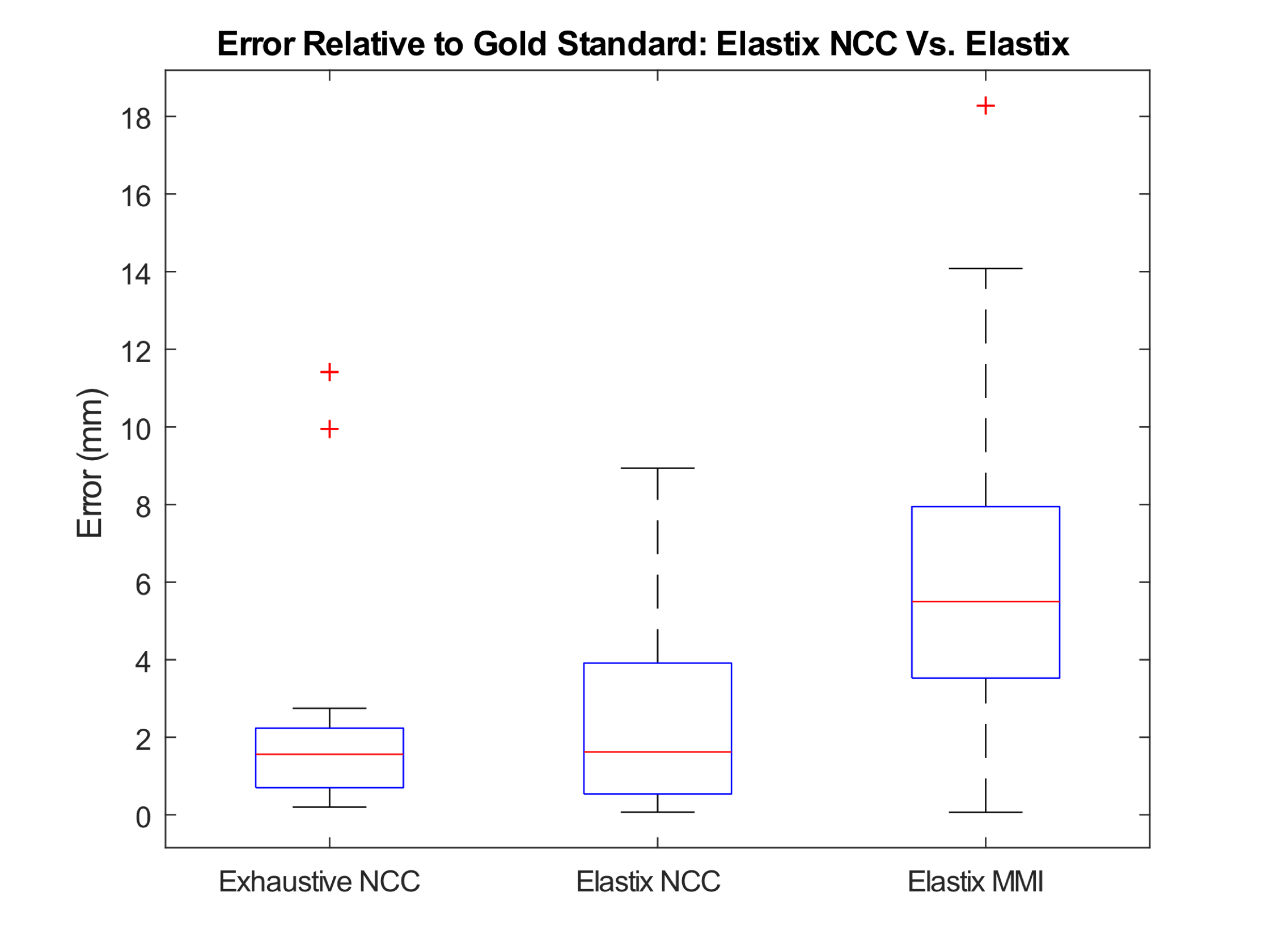


fig. 3 Boxplots illustrating difference in error distributions between algorithm using NCC and Elastix after optimisation.


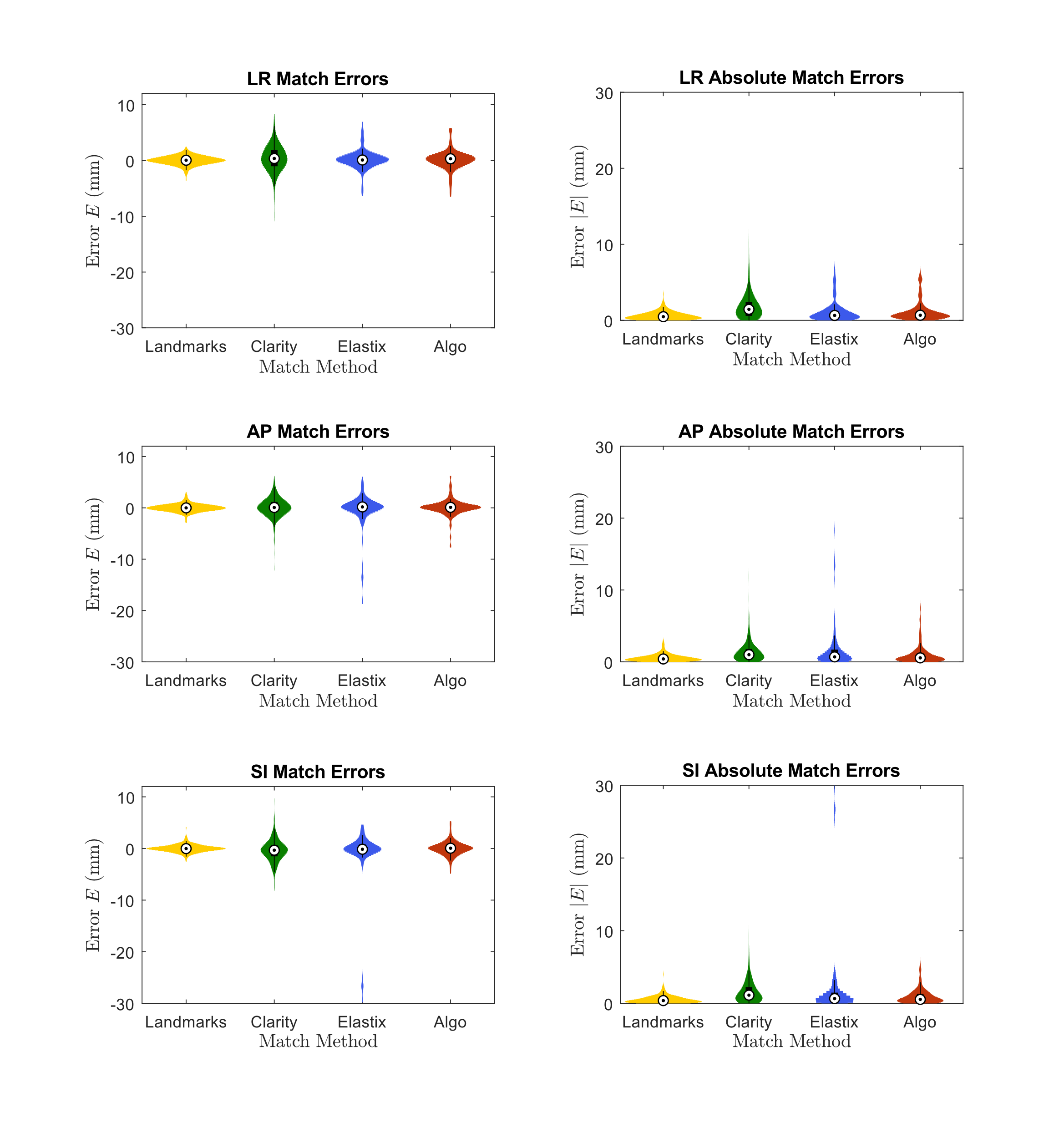


(b)

(d)

(f)

(e)

(c)

(a)

fig. 4 Error distributions of manual landmark matches, Clarity, Elastix and algorithm matches in Left-Right patient axes (a-b), Anterior-Posterior axes (c-d) and Superior-Inferior axes (e-f).
